# Measuring the Pull-Off Force of an Individual Fiber Using a Novel Picoindenter/Scanning Electron Microscope Technique

**DOI:** 10.3390/ma10091074

**Published:** 2017-09-13

**Authors:** Rahul Sahay, Ihor Radchenko, Arief S. Budiman, Avinash Baji

**Affiliations:** 1 Engineering Product Development (EPD) Pillar, Singapore University of Technology and Design, 8 Somapah Rd, Singapore 487372, Singapore; rahul_sahay@sutd.edu.sg; 2 The Xtreme Materials Laboratory (XML), Singapore University of Technology and Design, 8 Somapah Rd, Singapore 487372, Singapore; ihor_radchenko@mymail.sutd.edu.sg

**Keywords:** picoindenter, SEM, pull-off force, mechanical properties, electrospinning

## Abstract

We employed a novel picoindenter (PI)/scanning electron microscopy (SEM) technique to measure the pull-off force of an individual electrospun poly(vinylidene fluoride) (PVDF) fibers. Individual fibers were deposited over a channel in a custom-designed silicon substrate, which was then attached to a picoindenter. The picoindenter was then positioned firmly on the sample stage of the SEM. The picoindenter tip laterally pushed individual fibers to measure the force required to detach it from the surface of substrate. SEM was used to visualize and document the process. The measured pull-off force ranged between 5.8 ± 0.2 μN to ~17.8 ± 0.2 μN for individual fibers with average diameter ranging from 0.8 to 2.3 μm. Thus, this study, a first of its kind, demonstrates the use of a picoindenter to measure the pull-off force of a single micro/nanofiber.

## 1. Introduction

Nanostructured materials and composites are extensively investigated for novel and functional applications due to their unique physical, chemical, and mechanical properties [1,2,3,4]. For example, reduced graphene oxide films are deposited and used as flexible and transparent semiconductors [1]. Similarly, nanowires and arrays of nanocantilevers are being extensively investigated for the detection of malignant lesions from biological fluids [2]. One such nanoscale material is organic/inorganic nanofibers, which has been widely employed in applications such as filtration, Li-ion batteries, nanoelectronics, bioinspired adhesives, and tissue engineering [5,6,7,8]. Techniques that are used to fabricate these nanofibers include electrospinning and melt-electrospinning [9,10]. Mechanical properties of these fibers are important in order to design fibrous membranes for applications such as scaffolds, wound dressings, filtration, and drug delivery [6,11,12,13,14]. For example, electrospun membranes have found applications as wound dressing materials, as these membranes can be elastic, flexible, and sufficiently strong [12]. Typically, the diameter of these electrospun fibers ranges from ~50 nm to few microns. Handling of these small-size fibers makes it difficult to test the mechanical properties of an individual fiber. Investigating the mechanical properties of an individual fiber is crucial and is required to develop models that can be used to understand the behavior of the membranes.

A variety of techniques have been used to measure the mechanical properties of individual fibers. These techniques include a method in which an individual fiber is attached to a cantilever tip of an atomic force microscope (AFM). The AFM cantilever is then used to bend the fiber to measure its Young’s modulus [15]. Another technique uses a tipless AFM cantilever to bend an individual electrospun fiber collected on a patterned substrate to measure its mechanical properties [16]. For example, Persano et al. [17] collected individual suspended PVDF fiber and measured the piezoelectric response of an individual fiber by applying mechanical stress using an indenter. One of the extensively used technique uses a combination of atomic force microscope and inverted optical microscope [18,19]. Individual electrospun fibers collected on a patterned substrate are mechanically operated with an AFM cantilever tip, and the process is visualized through an optical microscope. In order to investigate the mechanical properties of individual fibers in a compressive loading state, micro-/nano-pillar compression and the use of a nanoindenter (or picoindenter) is more common where the height-to-diameter ratio of the pillar is at least three [20,21,22,23].

In many of the AFM-related techniques, the AFM tip is used to stretch the fiber along the patterned substrate. The load vs. displacement curves thus collected are used to estimate the properties of individual electrospun fibers. The limitation associated with this technique is that the load can only be measured between 1 nN to 1 μN and the displacement is restricted between 10 pm to 10 μm. In this study, we use a picoindenter to measure the adhesion behavior of a single fiber in a scanning electron microscope (SEM). The technique offers advantages such as wide range of applied forces and displacements. Since the test is performed in-situ in a SEM, it enables us to visualize the detachment of the fiber from the substrate during the test. The applied forces in this technique can range from ~0.1 μN to 100 μN, and the displacements can range from ~0.1 nm to 1 mm. This increase of the measurement range is crucial and has proven useful in the measurement of strong adhesion force as will be evident later in the present study. Since the test is performed in a SEM, the detachment of the fiber can be clearly and precisely visualized compared to other optical techniques.

This manuscript particularly focuses on the pull-off force, the force required to detach the fiber from the surface of substrate. Pull-off force essentially measures the van der Waals forces between the fiber and the substrate. Pull-off force is essential for applications where organic/inorganic material attaches to the fibrous membrane under the action of van der Waals forces. These applications include tissue engineering (adhesion of cells to the fibers), nanocomposite (adhesion of nanoparticles, or adhesion of supporting matrix to the fibers), and adhesive membrane (adhesion of fibers to a given surface) [24,25,26,27,28,29]. In this study, pull-off force of individual electrospun PVDF fibers is measured with respect to monocrystalline silicon substrate (100). PVDF is chosen in this study because of its good processability, chemical inertness, mechanical flexibility, and its ability to display piezoelectric behavior. In our previous studies [27,28,29], we used electrospinning to obtain PVDF fibers and demonstrated that these PVDF fibers can be used for dry-adhesive applications. However, the adhesion behavior of a single PVDF fiber was not investigated in our previous studies.

## 2. Materials and Methods

**Figure 1:**
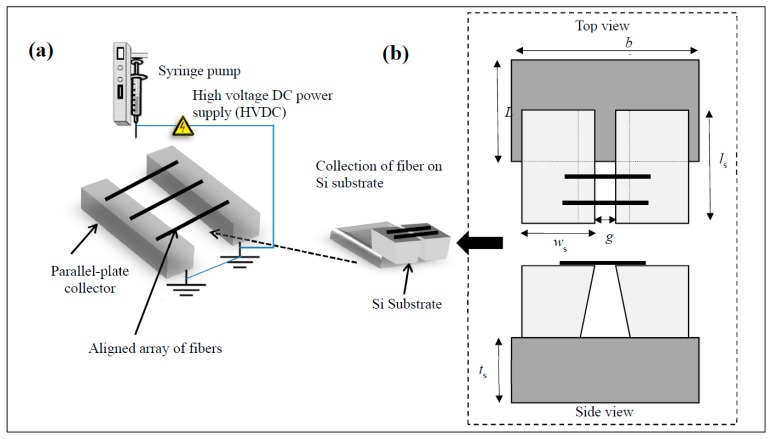
(**a**) Schematic of the electrospinning setup employed to collect uniaxially aligned poly(vinylidene fluoride) (PVDF) electrospun fibers; (**b**) schematic of the custom designed substrate employed to collect individual electrospun fibers. Here, *w*_s_ ~ 5 mm, *l*_s_ ~ 10 mm, *t*_s_ ~ 0.5 mm, *b* ~ 12 mm, and *L* ~ 10 m.

Polymer solutions were prepared by dissolving poly(vinylidene fluoride) (PVDF, M_W_ ~360,000) (Sigma Aldrich, Singapore) in dimethylformamide (DMF, Sigma Aldrich, Singapore) and acetone mixture in 1:2 (vol/vol) ratio. The concentration of PVDF in this solvent mixture was varied from 6.5 to 26 wt %. The concentration of PVDF in the solution was varied to collect fibers with diameters ranging from 0.8 to 2.3 μm. Following this, the polymer solution was fed into a syringe equipped with an 18 gauge needle. A parallel-plate collector was used to collect uniaxially aligned PVDF fibers as shown in Figure 1a [30]. Applied electric field during the electrospinning and the flow rate were maintained at ~1.0 kV/cm and ~0.5 mL/h, respectively.

Electrospun fibers were then collected on the custom-designed substrate for pull-off force measurements (see Figure 1b). The custom-designed substrate consists of three pieces of silicon wafers that were glued to each other with silver paste, as shown in Figure 1b. The dimensions of two identical silicon pieces were 5 mm × 10 mm × 0.5 mm (width × length × thickness). The dimensions of the third silicon piece were 12 mm × 10 mm × 0.5 mm (width × length × thickness). The typical width of the channel (*g*) used was ~0.5 mm. Individual electrospun fibers were then deposited over the channel in the custom designed substrate as shown in the schematic (Figure 1).

## 3. Results and Discussion

A scanning electron microscope (SEM) (JSM-6700F, JEOL, Peabody, MA, USA) was used to examine the morphology of the PVDF fibers. The diameter of the fibers was determined from these SEM images. SEM was also used to visualize the fiber orientation with respect to the substrate and to record the measurements.

The mechanical tests on these PVDF fibers were performed using a PI85 picoindenter (Hysitron, Eden Prairie, MN, USA) mounted directly onto an SEM stage. The Performech^®^ II advanced control module attached to picoindenter was used to control the x-, y-, and z-movement of the tip, while observing it under SEM. This in situ nanomechanical testing technique inside a SEM with high precision (in terms of loading as well as displacements) has been a recent development in the Xtreme Materials Laboratory (XML) at the Singapore University of Technology and Design (SUTD) [31]. Related nanomechanical testing techniques have also been developed to enable in situ fracture observation in small-scale samples and nanoscale interfacial adhesion strength of advanced, novel multilayer materials [32].

In the first step, the indenter tip is brought perpendicular to the axis of the fiber. The indenter tip is then raised and brought in contact with the fiber. The point of contact is *g*/2, i.e., the center of the channel of the substrate. The fiber is then pushed by the indenter tip until it delaminate from the substrate (see video 1). Following this, the tip is retracted back to bring the fiber back to its initial position. The tip displacement rate is constant, but varied from 100 nm/s to 1 μm/s for different fibers. SEM recorded the measurements at 40 frames/s. We estimate the errors in recording the pull-off force and fiber dimensions to be ±0.2 μN and ±200 nm, respectively.

**Figure 2:**
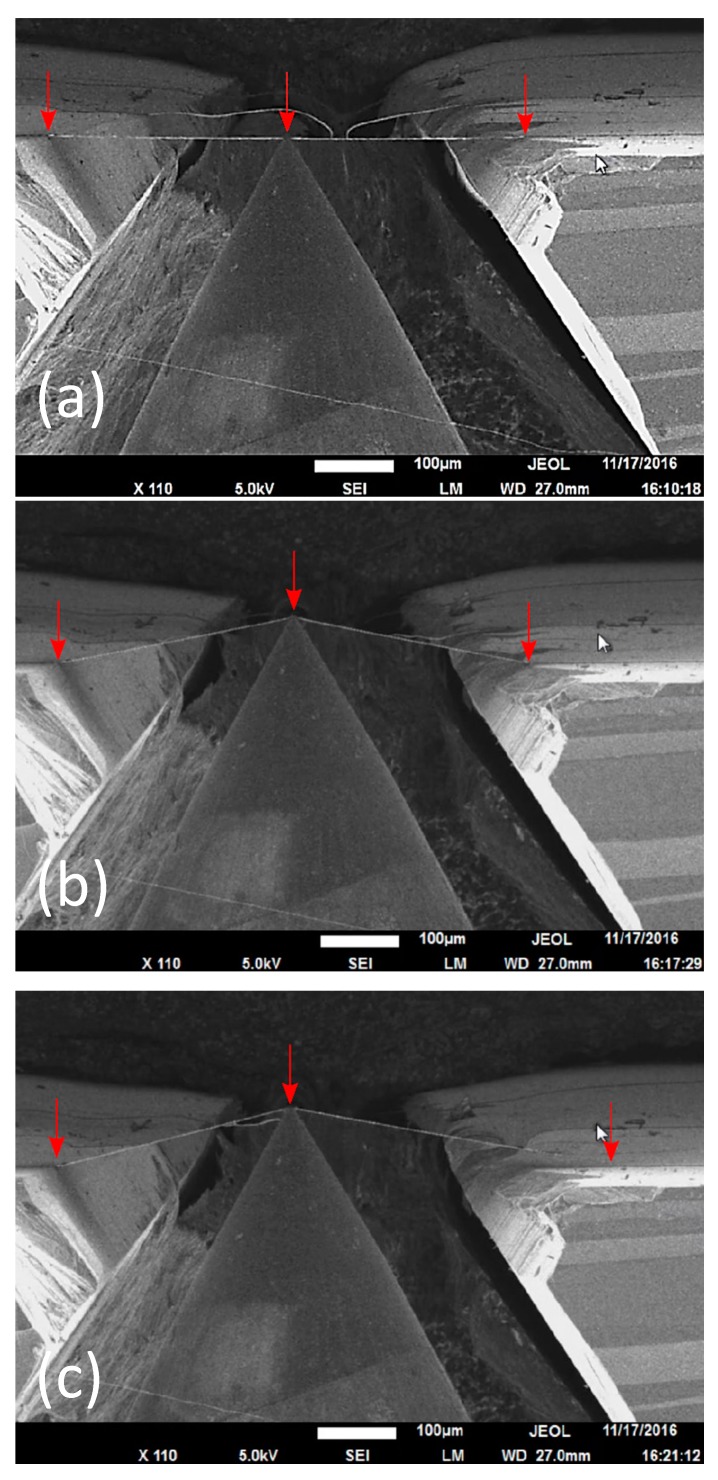
Scanning electron microscopy (SEM) movie frames depicting the delamination of individual PVDF electrospun fiber from silicon substrate. After the initial contact of the fiber with indenter tip (**a**); the fiber is stretched (**b**) and delaminated from substrate (**c**). Red arrows indicate the contact points of the fiber and substrate/indenter tip.

**Figure 3:**
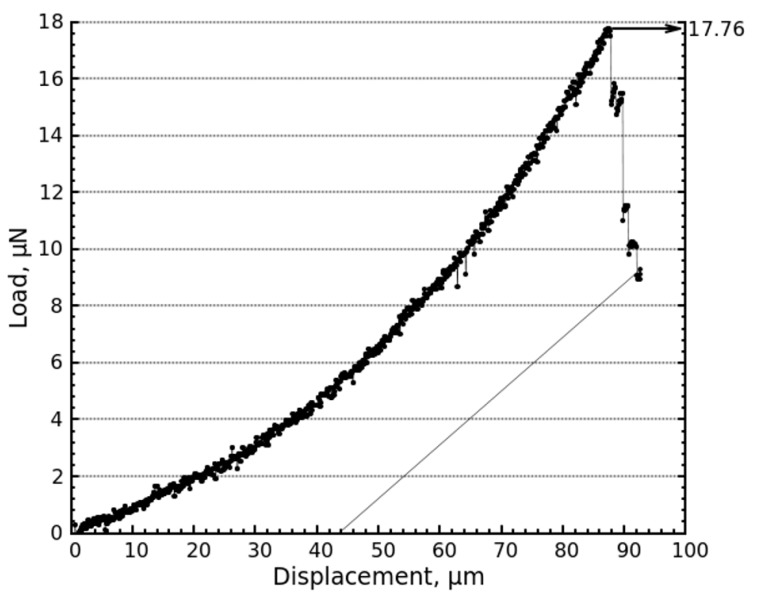
Representative load vs. displacement curve depicting pull-off force required to delaminate an individual PVDF fiber from silicon substrate.

Electrospun PVDF fibers deposited on the custom-designed substrate are seen to attach firmly to the substrate, as confirmed by the images collected during the measurements (see Figure 2). The adhesion of the fibers to the substrate is attributed to the van der Waals forces. An individual fiber bridging the channel is selected and pulled laterally at a constant displacement rate perpendicular to the surface of substrate until it delaminates from the surface. The tip is then retracted to bring the fiber back to its initial original unstretched position to complete the test cycle. SEM images taken during the test are shown in Figure 2. A representative load vs. displacement curve is plotted in Figure 3.

The pull-off force of fibers with diameters varying from 0.8 to 2.3 μm are measured from their respective load vs. displacement curves. Table 1 shows the values of pull-off force measured for fibers with diameters ranging from 0.8 to 2 μm. Pull-off force as high as ~17.8 ± 0.2 μN is obtained for fiber with ~1 μm diameter. The pull-off force varies from 5.83 ± 0.2 to 17.8 ± 0.2 μN for ~1 μm diameter fiber. The variation in pull-off force for a given fiber diameter (~1μm) can be attributed to (a) variation in the width (*g*) of the channel of silicon substrate; (b) mode of delamination of fiber from the surface of the substrate, which can be intermittent or continuous; and (c) orientation of the fiber with respect to the picoindenter tip. Further work is on-going to quantify the pull-off force as a function of all governing parameters. This future study will help in developing design-maps to predict pull-off force of single fibers with respect to the governing parameters. This, in turn, will help in predicting the properties of membranes composed of these single fibers.

## 4. Conclusions

We present a preliminary study on measuring the adhesion properties of individual electrospun fibers using a novel technique combining picoindenter and scanning electron microscopy. This technique can be used to determine the mechanical property including adhesion behavior of an individual electrospun fiber, which will be useful to design macroscopic structures with desirable mechanical properties. Pull-off force of individual PVDF electrospun fibers from the silicon substrate was determined. Pull-off forces ranging between 5.8 ± 0.2 and 17.8 ± 0.2 μN were measured for individual electrospun PVDF fibers with an average diameter ranging from 0.8± 0.2 μm to 2.3 ± 0.2 μm. Further studies are under way to understand the interaction between individual fibers (varying compositions and varying diameters,) with varieties of substrates, under different operating conditions.

## Figures and Tables

**Table 1:** Pull-off force measured for fibers with diameters ranging from 0.8 to 2 μm.

S. No	Fiber Diameter (μm)	Pull-Off Force (μN)
1	0.8	5.5 ± 0.2
2	1	5.83 ± 0.16
3	1	17.76 ± 0.16
4	1.4	7.3 ± 0.2
5	2	8.93 ± 0.16
6	2	12.17 ± 0.5

## Supplementary Materials

The following are available online at www.mdpi.com/1996-1944/10/9/1074/s1.

## References

[1] Eda G., Fanchini G., Chhowalla M. (2008). Large-area ultrathin films of reduced graphene oxide as a transparent and flexible electronic material. Nat. Nanotechnol..

[2] Ferrari M. (2005). Cancer nanotechnology: Opportunities and challenges. Nat. Rev. Cancer.

[3] Maynard A.D., Aitken R.J., Butz T., Colvin V., Donaldson K., Oberdörster G., Philbert M.A., Ryan J., Seaton A., Stone V. (2006). Safe handling of nanotechnology. Nature.

[4] Guo P. (2010). The emerging field of RNA nanotechnology. Nat. Nanotechnol..

[5] Burger C., Hsiao B.S., Chu B. (2006). Nanofibrous Materials and Their Applications. Annu. Rev. Mater. Res..

[6] Smith L.A., Ma P.X. (2004). Nano-fibrous scaffolds for tissue engineering. Colloids Surf. B Biointerfaces.

[7] Kumar P.S., Sahay R., Aravindan V., Sundaramurthy J., Ling W.C., Thavasi V., Mhaisalkar S.G., Madhavi S., Ramakrishna S. (2012). Free-standing electrospun carbon nanofibres—A high performance anode material for lithium-ion batteries. J. Phys. D Appl. Phys..

[8] Sahay R., Low H.Y., Baji A., Shaohui F., Wood K.L. (2015). A State-of-the-Art Review and Analysis on the Design of Dry Adhesion Materials for Applications such as Climbing Micro-robots. RSC Adv..

[9] Baji A., Mai Y.-W., Wong S.C. (2015). Effect of fiber size on structural and tensile properties of electrospun polyvinylidene fluoride fibers. Polym. Eng. Sci..

[10] Góra A., Sahay R., Thavasi V., Ramakrishna S. (2011). Melt-Electrospun Fibers for Advances in Biomedical Engineering, Clean Energy, Filtration, and Separation. Polym. Rev..

[11] Lee S., Chong S.Y.C., Tuck S.J., Corey J.M., Chan J.R. (2013). A rapid and reproducible assay for modeling myelination by oligodendrocytes using engineered nanofibers. Nat. Protoc..

[12] Vargas E.A.T., Baracho N.C.d.V., de Brito J., de Queiroz A.A.A. (2010). Hyperbranched polyglycerol electrospun nanofibers for wound dressing applications. Acta Biomater..

[13] Laforgue A., Robitaille L. (2010). Production of conductive PEDOT nanofibers by the combination of electrospinning and vapor-phase polymerization. Macromolecules.

[14] Mickova A., Buzgo M., Benada O., Rampichova M., Fisar Z., Filova E., Tesarova M., Lukas D., Amler E. (2012). Core/shell nanofibers with embedded liposomes as a drug delivery system. Biomacromolecules.

[15] Gu S.Y., Wu Q.L., Ren J., Vancso G.J. (2005). Mechanical properties of a single electrospun fiber and its structures. Macromol. Rapid Commun..

[16] Yang L., Fitié C.F.C., van der Werf K.O., Bennink M.L., Dijkstra P.J., Feijen J. (2008). Mechanical properties of single electrospun collagen type I fibers. Biomaterials.

[17] Persano L., Catellani A., Dagdeviren C., Ma Y.J., Guo X.G., Huang Y.G., Calzolari A., Pisignano D. (2016). Shear piezoelectricity in poly(vinylidenefluoride-co-trifluoroethylene): Full piezotensor coefficients by molecular modeling, biaxial transverse response, and use in suspended energy-harvesting nanostructures. Adv. Mater..

[18] Baker S.R., Banerjee S., Bonin K., Guthold M. (2016). Determining the mechanical properties of electrospun poly-ε-caprolactone (PCL) nanofibers using AFM and a novel fiber anchoring technique. Mater. Sci. Eng. C.

[19] Carlisle C.R., Coulais C., Guthold M. (2010). The mechanical stress-strain properties of single electrospun collagen type I nanofibers. Acta Biomater..

[20] Uchic M.D. (2004). Sample Dimensions Influence Strength and Crystal Plasticity. Science.

[21] Budiman A.S., Han S.M., Greer J.R., Tamura N., Patel J.R., Nix W.D. (2008). A search for evidence of strain gradient hardening in Au submicron pillars under uniaxial compression using synchrotron X-ray microdiffraction. Acta Mater..

[22] Burek M.J., Budiman A.S., Jahed Z., Tamura N., Kunz M., Jin S., Han S.M.J., Lee G., Zamecnik C., Tsui T.Y. (2011). Fabrication, microstructure, and mechanical properties of tin nanostructures. Mater. Sci. Eng. A.

[23] Kim Y., Budiman A.S., Baldwin J.K., Mara N.A., Misra A., Han S.M. (2012). Microcompression study of Al-Nb nanoscale multilayers. J. Mater. Res..

[24] Baji A., Zhou L. (2015). On the Adhesion performance of a single electrospun fiber. Appl. Phys..

[25] Sahoo N.G., Rana S., Cho J.W., Li L., Chan S.H. (2010). Polymer nanocomposites based on functionalized carbon nanotubes. Prog. Polym. Sci..

[26] Martín J., Mijangos C. (2009). Tailored polymer-based nanofibers and nanotubes by means of different infiltration methods into alumina nanopores. Langmuir.

[27] Sahay R., Parveen H., Ranganath A.S., Ganesh V.A., Baji A. (2016). On the adhesion of hierarchical electrospun fibrous structures and prediction of their pull-off strength. RSC Adv..

[28] Sahay R., Parveen H., Baji A., Ganesh V. (2017). Fabrication of PVDF hierarchical fibrillar structures using electrospinning for dry-adhesive applications. J. Mater..

[29] Sahay R., Baji A., Ranganath A.S., Ganesh V.A. (2017). Durable adhesives based on electrospun poly(vinylidene fluoride) fibers. J. Appl. Polym. Sci..

[30] Sahay R., Thavasi V., Ramakrishna S. (2011). Design modifications in electrospinning setup for advanced applications. J. Nanomater..

[31] Xtreme Materials Laboraroty. http://xml.sutd.edu.sg/publications.

[32] Shivakumar R., Tippabhotla S.K., Handara V.A., Illya G., Tay A.A.O., Novoa F., Dauskardt R.H., Budiman A.S. (2016). Fracture Mechanics and Testing of Interface Adhesion Strength in Multilayered Structures—Application in Advanced Solar PV Materials and Technology. Procedia Eng..

